# *N*-Glycosylation of mollusk hemocyanins contributes to their structural stability and immunomodulatory properties in mammals

**DOI:** 10.1074/jbc.RA119.009525

**Published:** 2019-11-12

**Authors:** Michelle L. Salazar, José M. Jiménez, Javiera Villar, Maira Rivera, Mauricio Báez, Augusto Manubens, María Inés Becker

**Affiliations:** ‡ Fundación Ciencia y Tecnología para el Desarrollo (FUCITED), Santiago 7750269, Chile; § Departamento de Bioquímica y Biología Molecular, Facultad de Ciencias Químicas y Farmacéuticas, Universidad de Chile, Santiago 8380494, Chile; ¶ Departamento de Investigación y Desarrollo, Biosonda Corp., Santiago 7750269, Chile

**Keywords:** N-linked glycosylation, carbohydrate function, protein stability, humoral response, cytokine induction, glycoprotein, immunotherapy, mollusk hemocyanin, quaternary structure, secondary structure

## Abstract

Hemocyanins are widely used as carriers, adjuvants, and nonspecific immunostimulants in cancer because they promote Th1 immunity in mammals. Hemocyanins also interact with glycan-recognizing innate immune receptors on antigen-presenting cells, such as the C-type lectin immune receptors mannose receptor (MR), macrophage galactose lectin (MGL), and the Toll-like receptors (TLRs), stimulating proinflammatory cytokine secretion. However, the role of *N*-linked oligosaccharides on the structural and immunological properties of hemocyanin is unclear. Mollusk hemocyanins, such as *Concholepas concholepas* (CCH), *Fissurella latimarginata* (FLH), and *Megathura crenulata* (KLH), are oligomeric glycoproteins with complex dodecameric quaternary structures and heterogeneous glycosylation patterns, primarily consisting of mannose-rich *N*-glycans. Here, we report that enzyme-catalyzed *N*-deglycosylation of CCH, FLH, and KLH disrupts their quaternary structure and impairs their immunogenic effects. Biochemical analyses revealed that the deglycosylation does not change hemocyanin secondary structure but alters their refolding mechanism and dodecameric structure. Immunochemical analyses indicated decreased binding of *N*-deglycosylated hemocyanins to the MR and MGL receptors and TLR4 and reduced endocytosis concomitant with an impaired production of tumor necrosis factor α, and interleukins 6 and 12 (IL-6 and IL-12p40, respectively) in macrophages. Evaluating the function of *N*-deglycosylated hemocyanins in the humoral immune response and their nonspecific antitumor effects in the B16F10 melanoma model, we found that compared with native hemocyanins *N*-deglycosylated hemocyanins elicited reduced antibody titers, as well as partially diminished antitumor effects and altered carrier activities. In conclusion, the glycan content of hemocyanins is, among other structural characteristics, critically required for their immunological activities and should be considered in biomedical applications.

## Introduction

Mollusk hemocyanins are large oxygen-carrier glycoproteins present in the hemolymph of some mollusks. These glycoproteins are widely used in biomedicine and biotechnology because they induce Th1 immune responses when inoculated in mammals. Hemocyanins are used as carriers, adjuvants in cancer therapeutic vaccines based on dendritic cells, and nonspecific immunostimulants in the treatment of superficial bladder cancer, among other applications (1–3). Hemocyanin from the keyhole limpet (*Megathura crenulata*), known as KLH,^3^ is the most commonly used hemocyanin in the abovementioned applications (4). However, alternative gastropod hemocyanins with similar immunological properties, such as CCH from *Concholepas concholepas*, FLH from *Fissurella latimarginata*, HpH from *Helix pomatia*, RvH from *Rapana venosa*, HtH from *Haliotis tuberculata,* HlH from *Helix lucorum*, and HaH from *Helix aspersa,* among others, have been characterized (5–12).

Mollusk hemocyanins are high-molecular-weight oligomeric glycoproteins (4–8 MDa) characterized by a complex quaternary didecameric structure with repeated epitopes (3). These glycoproteins are composed of 10 subunits, which form hollow cylindrical structures known as decamers. Each subunit (350–450 kDa) has eight globular oxygen-binding domains, known as functional units (FUs), which are differentially glycosylated (13, 14). Additionally, gastropod hemocyanins are composed of one or two types of subunits associated noncovalently to form heterodidecameric structures, such as CCH, or homodidecameric structures, such as KLH and FLH (6, 14). Several studies using differential-scanning calorimetry have shown that hemocyanins exhibit considerable thermal stability (melting temperature in the range of 83–90 °C). Moreover, biophysical techniques suggest that hemocyanins are stable in a long range of pH values, which is probably a consequence of the interactions between subunits and the high degree of oligomerization that stabilize the quaternary structure (7, 8, 15, 16).

Another relevant characteristic of hemocyanins is their high-carbohydrate content, which comprises 3–4% (w/w) of the molecules, such as CCH and KLH; however, this value had not been determined until the present work for FLH. Hemocyanin *N*-glycosylation motifs are conserved near active sites and between binding sites of their subunits. These *N*-glycans participate in the assembly and stability of quaternary structures (17). Indeed, glycosylations increase protein solubility and prevent their denaturation or aggregation. Thus, glycans have been described as an essential post-translational modification in terms of protein stability (18). Besides, hemocyanin glycans are highly heterogeneous and primarily composed of mannose-rich *N*-glycans, as well as *N*-mixed carbohydrates with fucose, galactose, GlcNAc, and glycosylation branches that are not found in mammals (19–26). Furthermore, hemocyanins contain highly immunogenic glycan patterns, such as Fuc(α1–3)GalNAc(β1–4)[Fuc(α1–3)]GlcNAc, also observed in the human parasite *Schistosoma mansoni* (24), and Gal(β1–6) moieties, which have been found in some *O-*specific side chains of *Salmonella* lipopolysaccharides and capsular polysaccharides of *Klebsiella pneumoniae* (24, 25). Additionally, KLH contains Gal(β1–6)GalNAc, an immunogenic glycotope known as T antigen, which is present on some tumor cells (27). Interestingly, KLH and FLH, unlike CCH, exhibit exposed *O-*glycosylations and xylose. Therefore, it has been proposed that, in addition to their structural role, hemocyanin glycans could be highly immunogenic due to their heterogeneity and complexity (6, 14).

Previous data have shown that hemocyanin glycans, which act as multivalent ligands, mediate their interactions with several innate immune receptors on murine antigen-presenting cells (APCs), such as carbohydrate-recognizing C-type lectin receptors (CLRs) and Toll-like receptors (TLRs). In addition, CCH, FLH, and KLH bind *in vitro* to human mannose receptor (MR) and dendritic cell-specific intercellular adhesion molecule-3–grabbing nonintegrin (DC-SIGN) with high affinities in a glycan-dependent manner (28). Moreover, KLH binding to human MR triggers proinflammatory responses in APCs (29). Similarly, TLR4 has been shown to participate in the immunostimulatory effects of CCH, FLH, and KLH in murine APCs (30). All these receptors recognize glycosylated structures from pathogens and promote endocytosis, proinflammatory responses, and antigen presentation to T lymphocytes (31–33). Regarding hemocyanins, CCH, FLH, and KLH are incorporated by APCs by both macropinocytosis and receptor-mediated endocytosis and then slowly processed (34). Thus, hemocyanins undergo prolonged antigen presentation to T or B lymphocytes, promoting a Th1 immune response and the abovementioned antitumor effects. We have shown that hemocyanins promote the secretion of proinflammatory cytokines by APCs, such as TNFα, IL-6, and IL-12p40, with varying intensity and temporality for each hemocyanin (35).

In addition to the beneficial proinflammatory effects of hemocyanins as adjuvants in the early steps of immune responses (30), these glycoproteins have been shown to induce a potent humoral and cellular immune response, as well as an antitumor effect in mammals by themselves. Indeed, CCH showed comparable antitumor properties to KLH, whereas FLH showed superior properties to KLH in B16F10 murine melanoma models (6). Similarly, these hemocyanins displayed antitumor effects in models of superficial bladder cancer and oral cancer, as well as a carrier of a tumor associate mimotope of melanoma (36–39). Furthermore, RtH and HpH had potential antitumor effects in a murine model of colon carcinoma (40), and HaH showed antiproliferative effects in various carcinoma cell lines (41). Remarkably, previous results showed that the chemical deglycosylation of FLH significantly decreased its antitumor properties in a murine B16F10 melanoma model. Moreover, *in vitro* analyses of cytokine secretion by murine APCs showed that chemically-deglycosylated FLH induced a decreased amount of IL-6 and IL-12p40, suggesting that heterogeneous hemocyanin glycans might act as multivalent ligands and contribute to their extensive immune effects (6). Furthermore, shared glycan epitopes between hemocyanins and tumor cells induce cross-reacting antibodies, which promote antibody-mediated cellular cytotoxicity against tumors (25). However, despite all of these and other potential applications of hemocyanins, the mechanism by which they act as nonspecific immunomodulators as well as the role of *N*-glycans to these effects have not been comprehensively elucidated.

In this context, this study investigated whether enzymatic *N*-deglycosylation of CCH, FLH, and KLH with peptide:*N*-glycosidase F (PNGase F; EC 3.5.1.52) affects their structural, immunogenic, and nonspecific immunomodulatory properties. Hemocyanin residual glycans were analyzed by lectin array blotting, revealing a partial but significant glycan removal. Transmission EM (TEM) and size-exclusion chromatography (SEC) analyses showed that hemocyanin *N*-deglycosylation decreased their didecameric quaternary structure. Moreover, CD analyses showed that hemocyanin *N*-glycans contributed to the refolding of these proteins. Furthermore, we showed that *N*-glycans contributed to the recognition of the hemocyanins by different chimeric innate immune receptors, because ELISA analyses showed decreased binding of *N*-deglycosylated hemocyanins. In addition, analyses of J774.2 murine macrophages by flow cytometry and ELISA demonstrated decreased endocytosis of *N*-deglycosylated FLH and KLH, together with impaired production of proinflammatory cytokines. Finally, the immunogenic activity caused by *N*-deglycosylated hemocyanins was evaluated in the B16F10 murine melanoma model and showed a diminished specific humoral immune response against FLH and KLH, and a reduced nonspecific antitumor activity, compared with those of native hemocyanins. In addition, the carrier properties of *N*-deglycosylated hemocyanins were assessed in mice, coupling them with the hapten 1-fluoro-2,4-dinitrobenzene (DNFB), resulting in a decreased carrier effect for FLH. Based on these findings, we concluded that hemocyanin *N*-glycans are, among other structural characteristic, relevant for their structure and contribute to their immunogenicity and nonspecific immunomodulatory properties.

## Results

### Enzymatic hemocyanin N-deglycosylation resulted in a significant and partial removal of glycans

**Figure 1.**
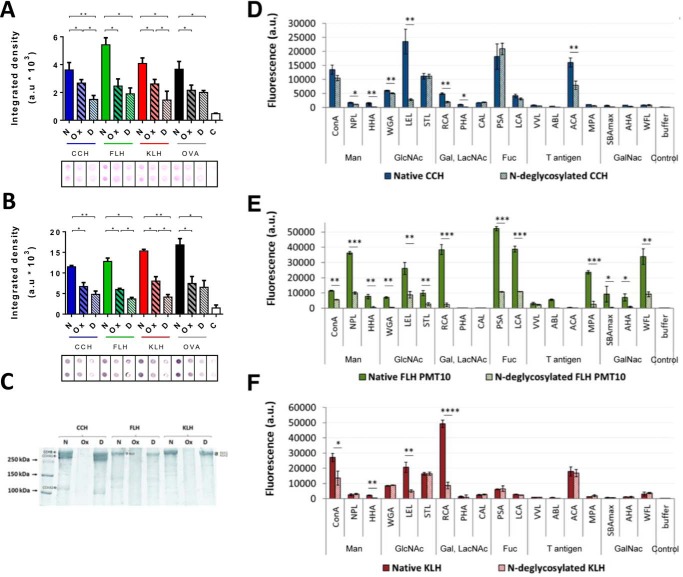
**PNGase F removed partially but significantly *N-*glycans from hemocyanins.** *A,* dot-blot analysis with PAS-staining method. Native (*N*), chemically deglycosylated (*Ox*), and enzymatically *N*-deglycosylated (*D*) hemocyanins (CCH, FLH, and KLH) were analyzed. *B,* dot-blot analysis with lectin staining. 2 μg/ml concanavalin A plus avidin–FAL 1:3000 and NBT-BCIP, which detect *N*-glycans, were used. *A* and *B*, for both analyses ovalbumin (*OVA*) was used as a deglycosylation control. *White bars* (*C*) represent a control of the membranes with all the reagents except hemocyanin. Densitometry quantification of blots was analyzed using ImageJ to quantify integrated density in arbitrary units (*a.u*). *Black borders* indicate the cut of dots from the original membrane. Data are shown as the mean ± S.E. Statistical analysis by *t* test. *, *p* < 0.05; **, *p* < 0.01. Representative images are of three independent experiments. *C,* SDS-PAGE analysis. Native (*N*), chemically deglycosylated (*Ox*), and *N*-deglycosylated (*D*) hemocyanins were analyzed. Representative gradient acrylamide gel (5–15%) was stained with Coomassie Blue. Molecular mass standards are indicated on the *left*. In addition, hemocyanin subunits are indicated: *CCH* (CCH-B, CCHA-1, and CCHA-2), *FLH* (a single band), and *KLH* (KLH1 and KLH2). Representative image of three independent experiments. Lectin array blotting of native and *N*-deglycosylated (*D*) CCH, (*E*) FLH, and (*F*) KLH. Hemocyanins were analyzed by a specific lectin assay for mannose (*Man*), *N*-acetylglucosamine (*GlcNAc*), galactose (*Gal*), *N*-acetyllactosamine (*LacNAc*), fucose (*Fuc*), T antigen, and *N*-acetylgalactosamine (*GalNAc*). Each Alexa Fluor-555–hemocyanin (0.08 mg/ml) was incubated for 1 h at 37 °C with the abovementioned lectins. The binding was quantified in arbitrary fluorescence units (*fluorescence a.u.*). Data are shown as the mean ± S.E. of one experiment with five replicates. Statistical analysis was done by one-way ANOVA, comparing native hemocyanins *versus N*-deglycosylated hemocyanins. *, *p* < 0.05; **, *p* < 0.01; ***, *p* < 0.001; ****, *p* < 0.0001.

Hemocyanins were enzymatically *N*-deglycosylated with PNGase F using dissociating conditions to give a better access to oligosaccharides. To verify the removal of *N-*glycans, as a first approach we analyzed the samples by dot blots with periodic acid-Schiff (PAS) staining, which revealed the presence of sugars in a nonspecific manner (Fig. 1*A*), and later with concanavalin A (ConA), which revealed the presence of mannose-rich *N-*glycans (Fig. 1*B*). Ovalbumin (OVA), a glycoprotein whose sugars have previously been characterized, was used as a control of the enzymatic activity to remove glycans (42). As an additional control, chemically deglycosylated hemocyanins were also analyzed, as this protocol was previously established by us to partially but significantly remove both *N-* and *O-*glycans from these proteins (34). The results of these analyses indicate a partial but significant deglycosylation by the enzymatic method.

In addition, to evaluate the purity and integrity of the protein samples used for further experiments, we analyzed native and deglycosylated hemocyanins by SDS-PAGE (Fig. 1*C*). The results showed characteristic bands for CCH (CCH-B at 350 kDa, CCH-A1 at 300 kDa, and CCH-A2 at 108 kDa), FLH (350 kDa), and KLH (KLH1 and KLH2 had a very-similar mass of ∼350 kDa) (5, 36). As reported previously with chemically-deglycosylated hemocyanins treated with sodium periodate, the quaternary structure is not affected (Fig. S1). However, SDS-PAGE analysis showed that periodate-treated hemocyanin did not enter in the resolving portion of the gel, an effect due to the formation of Schiff bases within hemocyanin molecules (34). *N-*Deglycosylated hemocyanins had similar bands to native hemocyanins, but there was a slight shift in migration, which was quantified to estimate the percentage of sugars removed (Table S1). The glycan percentages previously reported for CCH and KLH (3.1 and 3.4% w/w) and those obtained here (2.5 ± 0.4 and 2.3 ± 0.4% w/w) might be different because we achieved only partial removal of the *N-*glycans (14, 36). The glycan content of FLH has not been reported previously, and the results indicated that *N-*glycans comprised at least 4.1 ± 1.1% (w/w).

To complement the previous results, we performed specific analyses by lectin array blotting, which provides individual lectin-binding profiles for each fluorescently-tagged protein allowing us to directly obtain global glycomic profiling of *N-* and *O-*glycans in glycoprotein samples, without the need to release or purify glycan moieties as other procedures (43). For this purpose, the binding of native and *N-*deglycosylated hemocyanins to 48 different lectins that recognize the different conformation of diverse monosaccharides, as shown in Table S2, was determined on an arbitrary scale. Chemically-deglycosylated hemocyanins could not be analyzed by this method because sodium periodate treatment modifies the primary amines of the proteins, and fluorophores cannot bind in this condition (34). We observed that CCH (Fig. 1*D*) bound primarily to ConA, triticum vulgaris agglutinin, LEL, STL, RCA, PSA, lens culinaris agglutinin, and ACA with different intensities. This binding pattern, according to the specificity of each lectin (Table S2), indicates that CCH possesses primarily α-mannoses and complex GlcNAc structures, in addition to fucose attached to these monosaccharides, which are the major *N-*glycosylation components. *N-*Acetylactosamines, lactoses, and β-galactoses were also detected to a lesser extent, the latter being the main components of *O-*glycosylations or mixed *N-*glycosylations (20). In contrast, *N-*deglycosylated CCH showed decreased binding primarily to HHA, LEL, RCA, and ACA, indicating a partial removal of complex *N-*acetylglucosamines, *N-*acetylactosamines, and galactoses. However, the binding to ConA, STL, or PSA did not decrease, indicating the presence α-mannoses, *N-*acetylactosamines, and fucoses.

FLH bound to the abovementioned lectins, in addition to NPL, HHA, VFA, MPA, SBAmax, AHA, and WFL, suggesting that FLH possesses terminal mannoses, *N-*acetylglucosamines linked to α-mannoses, and GalNAc, in addition to the previously mentioned monosaccharides (Fig. 1*E*). Moreover, FLH was analyzed with a low-power laser system (PMT 10), as its binding to some lectins produced a saturation of the fluorescence signal. These results confirmed that FLH glycans are more heterogeneous and abundant than CCH and KLH glycans. The binding of *N-*deglycosylated FLH to all lectins showed a significant decrease, indicating that only remnant mannoses, fucoses, galactoses, and *N-*acetylgalactosamines were present.

KLH showed a similar binding pattern to CCH, and its higher content of galactose is remarkable (Fig. 1*F*). In contrast, *N-*deglycosylated KLH showed a decreased binding to ConA, LEL, and RCA, indicating a partial removal of *N-*acetylglucosamines, α-mannoses, *N-*acetylactosamines, and galactoses, as well as the presence of remnant glycans. Notably, CCH and FLH bound to specific lectins for the tumor antigen Thomsen-Friedenreich (Galβ1–3GalNAc), known as T antigen, which has been reported in KLH (27).

Our results confirm the differences in *N-*glycan content and heterogenicity among CCH, FLH, and KLH. Thus, we next investigated whether these glycans contribute to the preferred didecameric structure of the hemocyanins, as described for other glycoproteins (17).

### N-Glycans contributed to the quaternary structure of hemocyanins

**Figure 2.**
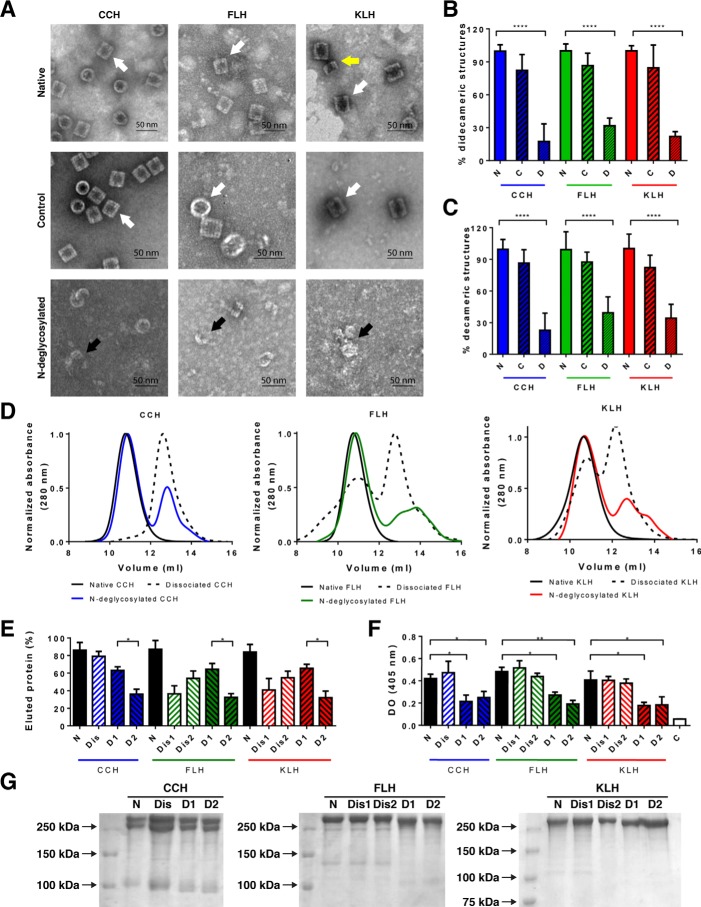
***N*-Glycans helped maintain the hemocyanin quaternary structure.** *A,* analysis by TEM of CCH, FLH, and KLH. Negative staining of native and *N*-deglycosylated hemocyanins and a control condition where hemocyanins underwent the *N*-deglycosylation protocol without the addition of PNGase F is shown. Representative images (lateral, *rectangles*; axial; *circles*; views of the hemocyanin molecules available on the grid) of two independent experiments of each hemocyanin at ×60,000, in which didecamers (*white arrows*), decamers (*yellow arrow*), and dissociated structures (*black arrows*) are observed. *Scale bar*: 50 nm. *B,* quantification of didecameric structures. 20 different photographs were quantified, where the didecamers of native hemocyanins were considered as 100%. *C,* quantification of decameric structures. A procedure similar to *B* was used. Statistical analysis by *t* test, comparing native *versus N*-deglycosylated hemocyanins. ****, *p* < 0.0001. *D,* size-exclusion chromatography of native, dissociated, and *N*-deglycosylated hemocyanins performed with a TSKgel® G5000PWXL column at 0.6 ml/min. Eluted proteins were monitored at 280 nm. *Black lines* are native hemocyanins; *dashed lines* are dissociated hemocyanins; and *colored lines* are *N*-deglycosylated hemocyanins. *E,* quantitative analysis of chromatographic peaks observed in *D*. The percentage of each species in the chromatograms was estimated according to the area of each peak using the molar extinction coefficient of 1.4 for CCH and 2.02 for FLH and KLH. Results from the first and the second dissociated peaks are indicated as 1 and 2 (*Dis1* and *Dis2*), respectively. Similarly, *N*-deglycosylated peaks are indicated as 1 and 2 (*D1* and *D2*), according to the first and the second peak. *F,* characterization of fractions by its *N*-glycan content. Eluted fractions from *D* were analyzed in ELISA plates by lectin staining (ConA plus avidin–FAL and pNPP). Plate optical density was read at 405 nm. Statistical analysis by *t* test, comparing native *versus N*-deglycosylated hemocyanins. *, *p* < 0.05; **, *p* < 0.01. *G,* SDS-PAGE analyses. Fractions eluted from *D* were analyzed. Representative gradient acrylamide gels (5–15%) were stained with Coomassie Blue. Molecular mass standards are indicated on the *left*. Data are representative of three experiments.

Previous experiments performed by Gai *et al.* (16) with the hemocyanin from the cephalopod *Todarodes pacificus* showed that the subunits from this protein decrease their capacity to form decameric oligomeric structures, after being *N-*deglycosylated using an equivalent protocol to the one used in our experiments. Hence, to determine whether the *N-*glycans have a role in the stability of quaternary hemocyanin structure, native and *N-*deglycosylated CCH, FLH, and KLH were analyzed by TEM using negative staining. Representative images of two experiments showed lateral (*rectangles*) and axial (*circles*) views of the didecameric hemocyanin molecules available on the grid in native samples and control samples without PNGase F, in contrast to mostly dissociated or incomplete decamers in *N-*deglycosylated samples (Fig. 2*A*). In addition, quantification of didecamers and decamers for each treatment in 20 representative images showed that the formation of both structures decreased in *N-*deglycosylated samples compared with the native samples (Fig. 2, *B* and *C*). Furthermore, the distribution of quaternary structures of native, dissociated, and *N-*deglycosylated hemocyanins was analyzed by SEC in a TSKgel G5000PWXL column suited for analyses of polymers up to 1 MDa. Fig. 2, *D* and *E,* shows the size-exclusion chromatograms and the quantitative analysis of the eluted species, respectively. Native hemocyanins showed a single peak of high molecular weight (10–12 ml of eluted volume). In the case of dissociated hemocyanins, which do not form didecamers as seen by TEM (Fig. S1), the elution profile of CCH (Dis-CCH) showed a single peak of lower molecular weight (12–14 ml of eluted volume), whereas FLH and KLH showed two peaks of high- and low-molecular weight nearly equally populated (Dis1 and Dis2 FLH and KLH). Thus, some dissociated species co-elute with didecameric structures. Indeed, as the column resolution limit is up to 1 MDa, and didecameric structure mass is around 8 MDa, some partially dissociated species bigger than monomers or dimers could not be separated from decamers by the column. The *N-*deglycosylated hemocyanins showed two elution peaks (D1 and D2), each one containing around 60 and 30% of the eluted protein, respectively. Overall, *N-*glycan removal induces the apparition of mixed species, which are partially dissociated structures over or below 1 Mda, and remarkably, they do not form the hollow cylinders as seen by TEM. Subsequently, the glycan content of each fraction was analyzed by ConA staining (Fig. 2*F*). The *N-*deglycosylated samples show a reduced glycan content, compared with native or dissociated hemocyanins, demonstrating that hemocyanin dissociation does not affect their binding to ConA. In addition, we observed no differences in the glycan content among each *N-*deglycosylated sample. Moreover, analyses of each eluted fraction by SDS-PAGE exhibited similar band patterns in native, dissociated, and *N-*deglycosylated proteins (Fig. 2*G*). Thus, differences in the elution pattern are due to the oligomerization of polypeptide chains.

These results support a role of *N-*oligosaccharides to stabilize and to hold the conformation of the didecameric quaternary structure of CCH, FLH, and KLH. Thus, we next investigated whether these *N-*glycans play a role in the secondary structure as well as in the folding process of hemocyanins.

### N-Deglycosylation did not change the secondary structure of hemocyanins but altered their refolding mechanism

**Figure 3.**
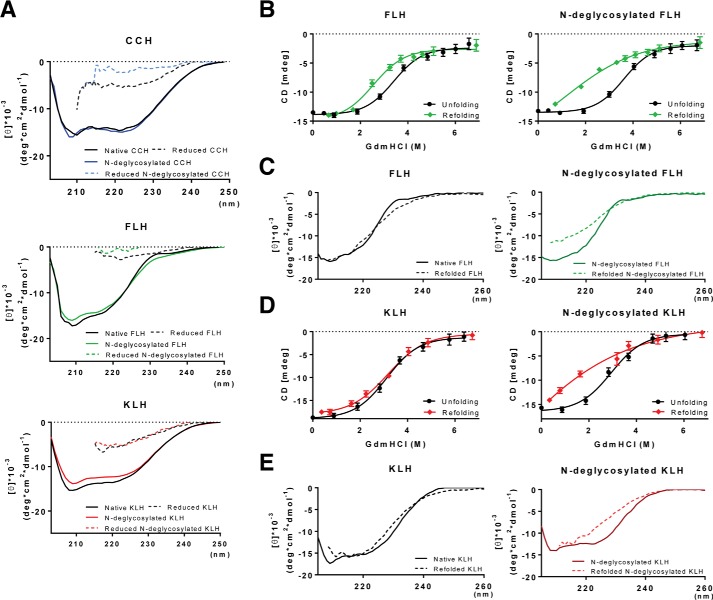
***N*-Deglycosylation did not modify the hemocyanin secondary structure but impaired hemocyanin refolding.** *A*, CD analyses. Native and *N*-deglycosylated hemocyanins in PBS or reducing conditions (2% β-mercaptoethanol) were analyzed by CD in the far-UV region (200–260 nm). *B,* unfolding and refolding curves of FLH. For the unfolding curves, native and *N*-deglycosylated FLH were treated with increasing concentrations (0–6.7 m) of GdmHCl for 24 h at room temperature. Similarly, for the refolding curves, samples previously treated with 6.7 m GdmHCl were diluted in PBS. Data are shown as the mean ± S.E. of at least 10 measurements of the dichroism signal at 222 nm from two independent experiments. *C,* comparison between the unfolded and refolded spectra of native and *N*-deglycosylated FLH. *D,* unfolding and refolding curves of KLH. Spectra of samples were obtained as described in *B. E,* comparison between the unfolded and refolded spectra of native and *N*-deglycosylated KLH. For all experiments, hemocyanins were analyzed at 0.25 mg/ml at 25 °C in a J-1500 JASCO spectropolarimeter. The CD spectra represent the average value of three measurements. In this case, the standard error is less than 10% for each wavelength determined. *B* and *C*, *errors bars* correspond to the variability of the CD signal observed during 30 s in two independent experiments.

To determine the effect of *N-*deglycosylation on the secondary structure of hemocyanins, CCH, FLH, and KLH were monitored by far-UV CD in native and reducing conditions (Fig. 3*A*). Results showed no differences between glycosylated and *N-*deglycosylated hemocyanin spectra, neither in native nor reduced conditions. Considerably, the KLH spectra are consistent with the results of previous studies (23), validating the results obtained here. Subsequently, chemical denaturation induced by guanidinium hydrochloride (Gdm-HCl) was used to determine the effect of *N-*deglycosylation on the protein stability. Guanidinium hydrochloride-induced unfolding curves of glycosylated and *N-*deglycosylated FLH and KLH, monitored by far-UV CD, were compared (Fig. 3, *B* and *D*). The unfolding of glycosylated and *N-*deglycosylated FLH and KLH occurs through a single sigmoid transition that is complete over 5 m Gdm-HCl. This process seems to be reversible for glycosylated hemocyanins because the intensity of the CD signal is recovered through a single sigmoid transition of refolding. For KLH, the refolding and unfolding transitions are almost superimposed (Fig. 3*D*), whereas FLH displays a hysteric behavior using similar incubation times with Gdm-HCl (Fig. 3*B*). This pattern of chemical stability is modified for *N-*deglycosylated hemocyanins. For *N-*deglycosylated FLH and KLH, the refolding seems incomplete because the native far-UV CD spectrum is not recovered upon refolding, whereas the glycosylated hemocyanins display a nearly superimposed CD spectrum (Fig. 3, *C* and *E*). These analyses could not be performed with CCH, because this protein is aggregated during the refolding process. Therefore, it is concluded that *N-*glycosylations contribute to the folding mechanism of FLH and KLH, but their presence is not relevant for the secondary structure once their structures are attained.

These results indicate the differences in the refolding processes of native and *N-*deglycosylated FLH and KLH, suggesting variations in the assembly of subunits to form homodidecameric structures. However, whether glycan content and heterogenicity correlate with the immunomodulatory properties of these glycoproteins has not been experimentally demonstrated. Thus, as a first approach to this question, we assessed whether *N-*deglycosylation modifies the binding of these hemocyanins to innate immune receptors *in vitro*.

### Hemocyanin N-glycans contributed to protein binding to innate immune receptors and promoted proinflammatory cytokine secretion

**Figure 4.**
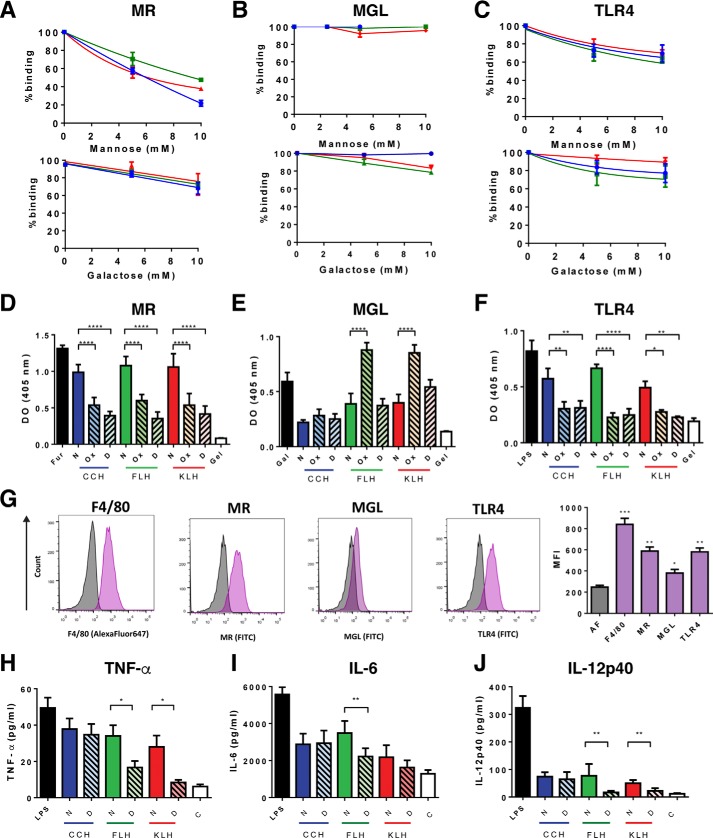
***N*-Glycans promoted hemocyanin binding to chimeric innate immune receptors and cytokine secretion in J774.2 macrophages.** Competitive hemocyanin binding to chimeric TLR4-Fc (*A*), MR-Fc (*B*), and MGL-Fc (*C*) in the presence of glycans, analyzed by ELISA. Chimeric receptors (1 μg/ml) were incubated with d-(+)-mannose or d-(+)-galactose (0–100 mm) and then with native hemocyanins (2.5 μg/ml). The binding was revealed with anti-Fc antibodies (goat anti-human Fc-FAL or goat anti-mouse Fc-FAL serum). *D–F*, reactivity of chimeric receptors with native (*N*), chemically deglycosylated (*Ox*), and *N*-deglycosylated (*D*) hemocyanins by ELISA. Hemocyanins (2.5 μg/ml) were incubated with the chimeric receptors (1 μg/ml) and with anti-Fc antibodies. Furfurman (*Fur*), lipopolysaccharide from *E. coli* (*LPS*), and d-(+)-galactose (*Gal*) were used as positive controls, and gelatin (*Gel*) was the negative control. *G,* analysis of innate immune receptors in J774.2 cells by flow cytometry. J774.2 cells were incubated with an anti-F4/80–Alexa Fluor-647 antibody, as well as primary anti-MR, anti-TLR-4, and anti-MGL following goat anti-rat IgG–FITC serum. Representative histograms of autofluorescence (*gray*) and the expression of the different markers (*purple*), as well as the quantification of the MFI are shown, including the autofluorescence signal (*AF*). Analysis of cytokine expression in J774.2 cells by ELISA. TNFα (*H*), IL-6 (*I*), and IL-12p40 (*J*) from J774.2 culture supernatants were quantified by ELISA after 24 h of incubation with native (*N*) and *N*-deglycosylated (*D*) hemocyanins. LPS from *E. coli* and the culture medium without hemocyanin (*C*) were used as positive and negative controls, respectively. For all experiments, the data are shown as the mean ± S.E. of three independent experiments. Analyses were done by *t* test. *, *p* < 0.5; **, *p* < 0.01; ***, *p* < 0.001; ****, *p* < 0.0001.

As previous studies have shown that hemocyanins bind to innate immune receptors of the C-type lectin (MR and MGL) and Toll-like receptor (TLR4) families with high affinity and in a glycan-dependent manner (28, 30), we assessed the contribution of hemocyanin mannose-rich *N-*glycosylations to their binding to chimeric MR-Fc, MGL-Fc, and TLR4-Fc. As a first approach, we analyzed the binding of native hemocyanins to MR-Fc, MGL-Fc and TLR4-Fc in the presence of increasing concentrations of mannose and galactose (0–10 mm), used as competitive ligands for these receptors. Results showed that MR binding to CCH, FLH, and KLH was mostly impaired by mannose and not by galactose (Fig. 4*A*). In contrast, mannose had no effect on hemocyanin binding to MGL, whereas galactose partially decreased FLH and KLH binding to this receptor (Fig. 4*B*). For TLR4, both mannose and galactose partially inhibited their binding to hemocyanins (Fig. 4*C*). Moreover, to reinforce these data, we compared the binding of these chimeric receptors to native and both chemically deglycosylated and enzymatically *N-*deglycosylated hemocyanins. We observed that native hemocyanins bound to MR (Fig. 4*D*), MGL (Fig. 4*E*), and TLR4 (Fig. 4*F*) in a similar manner to their respective positive controls (furfurman, galactose, and LPS). However, the binding of chemically deglycosylated or *N-*deglycosylated hemocyanins decreased significantly for all of them in their binding to MR and TLR4 but not for MGL. Thus, these assays show the differences between hemocyanins in their binding to these receptors. Indeed, MGL presents a low binding to native FLH and KLH, whereas CCH, either native or deglycosylated, did not bind this receptor (Fig. 4*E*). Instead, *N-*deglycosylated FLH and KLH binding to MGL was similar to the binding of native proteins. Strikingly, chemically-deglycosylated FLH and KLH showed substantial binding to MGL, which was significantly higher than that of the native proteins. This finding might be due to the partial removal of glycans, which could expose hidden galactoses from the core of the protein (44). To verify that all these differences are due to the glycan removal and not due to changes in the quaternary structure of hemocyanins, we compared the binding of the mentioned receptors to native and dissociated proteins, and no significant differences were observed (Fig. S2). Hence, these results showed that mannose- and galactose-rich *N-*glycosylations take part in the hemocyanin binding to several innate immune receptors *in vitro* (44).

The activation of innate immune receptors induced by hemocyanins promotes several responses in APCs, such as proinflammatory cytokine secretion (30, 35). Thus, we assessed whether hemocyanin *N-*deglycosylation, which impaired hemocyanin binding to immune receptors, influenced cytokine secretion. We used the J774.2 macrophage cell line as a suitable model for our studies because we confirmed that these cells expressed innate immune receptors such as MR, MGL, and TLR4 (Fig. 4*G*). Thus, J774.2 cells were incubated with native or *N-*deglycosylated hemocyanins for 24 h according to a previous work (35), and since at this time we observed a major cytokine response in kinetic curves (Fig. S3). Cytokines were quantified from culture supernatants. The results showed that hemocyanins induced TNFα production, which significantly decreased in cells treated with *N-*deglycosylated FLH and KLH (Fig. 4*H*). Subsequently, hemocyanins induced high levels of IL-6, whereas *N-*deglycosylated FLH reduced the production of this cytokine (Fig. 4*I*). Finally, significant production of IL-12p40 was observed when cells were treated with the three native hemocyanins, whereas *N-*deglycosylated FLH and KLH induced diminished levels of this cytokine (Fig. 4*J*). In contrast, *N-*deglycosylated CCH did not result in decreased cytokine production in any of these experiments. To verify that these results are due to deglycosylation and not to dissociation, we compared the cytokine levels induced by native and dissociated proteins, and no significant differences were observed (Fig. S2). Thus, despite the similarities in the structure and glycans of hemocyanins, their differences might provide specific recognition glycotopes that influence variable immune responses, such as incorporation by APCs (32), which was assessed after hemocyanin *N*-deglycosylation.

### Hemocyanin mannose-rich glycans contributed to their binding and incorporation by J774.2 cells

**Figure 5.**
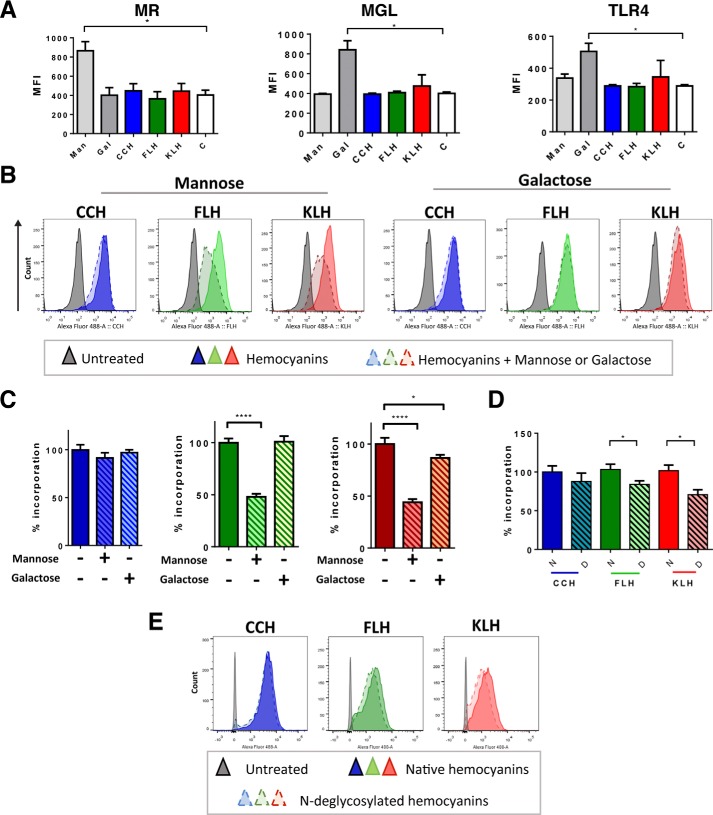
***N*-Glycans contributed to hemocyanin binding and incorporation by J774.2 macrophages.** *A,* effect of monosaccharides and CCH, FLH, and KLH on the expression of innate immune receptors analyzed by flow cytometry. J774.2 cells were treated with hemocyanins (50 μg/ml), mannose (*Man*), or galactose (*Gal*) (100 mm) for 1 h, and then incubated with an anti-F4/80–Alexa Fluor-647 antibody, as well as primary anti-MR, anti-TLR-4, and anti-MGL following goat anti-rat IgG–FITC serum. *Graphs* show the MFI of each sample. *B,* binding/incorporation of hemocyanins to J777-4 cells in the presence of monosaccharides. Representative histograms of J774.2 cells incubated with Alexa Fluor 488–hemocyanins (50 μg/ml) for 1 h in the presence or absence of mannose and galactose (100 mm). *C,* quantification of the data presented in *B*. The binding and incorporation of the abovementioned histograms, with the mean fluorescence intensity of cells with hemocyanins and without monosaccharides were defined as 100%. *D,* binding/incorporation of native and *N*-deglycosylated hemocyanins. Quantification of the binding and incorporation of hemocyanins, where the mean fluorescence intensity of native hemocyanins was defined as 100%. *E,* binding of native and *N*-deglycosylated fluorescent hemocyanins. Representative histograms of *D*. For all experiments, the viability was assessed using eFluor 780 dye. The data are presented as the mean ± S.E. of four independent experiments. Analysis was by *t* test. *, *p* < 0.05; and ****, *p* < 0.0001.

To determine whether mannose-rich glycans contribute to hemocyanin binding and incorporation in APCs, we performed flow cytometric analyses of J774.2 macrophages. Cells were preincubated with d-(+)-mannose and d-(+)-galactose and were subsequently incubated with Alexa Fluor-488–hemocyanins and with eFluor780 dye to select viable cells for further analyses. First, we verified the expression of MR, MGL, and TLR4 on macrophages after each stimulus. Results showed that mannose induced MR, whereas galactose increased MGL and TLR4 levels; however, hemocyanins did not enhance the levels of these receptors by themselves under these experimental conditions (Fig. 5*A*). Moreover, we observed that the binding and incorporation of Alexa Fluor-488–CCH was not inhibited by any of the sugars (Fig. 5, *B* and *C*). In contrast, the binding and incorporation of Alexa Fluor-488–FLH and Alexa Fluor-488–KLH were significantly inhibited by mannose (∼50% inhibited) (Fig. 5, *B* and *C*). Similarly, Alexa Fluor-488–KLH incorporation was reduced in the presence of galactose, although this inhibition was smaller (5%) (Fig. 5*C*). Next, we analyzed the binding and incorporation of Alexa Fluor-488–*N-*deglycosylated hemocyanins. For this experiment, J774.2 macrophages were pretreated with *N,N*-dimethyl amiloride (DMA) to inhibit nonspecific incorporation by macropinocytosis. No significant difference was observed in the mean fluorescence intensity (MFI) of macrophages treated with Alexa Fluor-488–native CCH and Alexa Fluor-488–*N-*deglycosylated CCH (Fig. 5, *D* and *E*). However, the binding and incorporation of Alexa Fluor-488–*N-*deglycosylated FLH and KLH decreased (∼10–20%) compared with that of native hemocyanins.

The results confirm that the variation in oligosaccharide epitopes induces different immune responses, as partial *N-*deglycosylation diminished the immunomodulatory properties of FLH and KLH but had no effect on CCH. Nevertheless, these experiments were performed in a cellular context *in vitro*. Thus, to assess the overall effect of hemocyanin *N-*glycans, we performed an *in vivo* experiment measuring humoral and antitumor responses in a murine melanoma model.

### N-Glycans contributed to hemocyanin-induced humoral response, carrier effect, and antitumor responses

**Figure 6.**
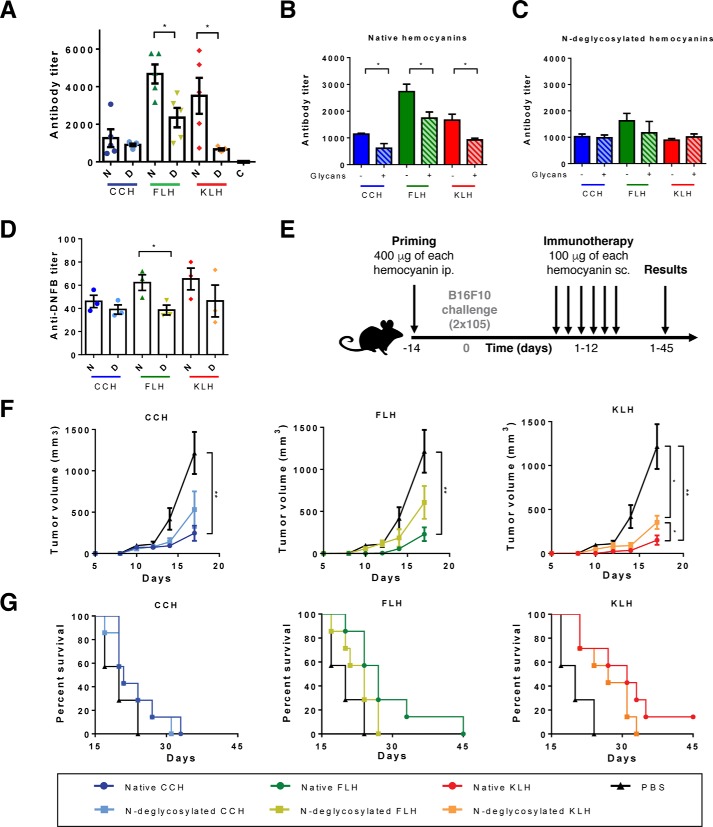
**Hemocyanin *N*-deglycosylation affected the humoral response, carrier properties, and antitumor activity.** *A,* effect of *N*-deglycosylation on the humoral immune response. The specific serum antibody titers of C57BL/6 mice that were immunized with 1 mg of native (*N*) or *N*-deglycosylated (*D*) hemocyanins were determined at day 45. Antibody titers against each hemocyanin form (native and *N*-deglycosylated) were estimated by ELISA, using an anti-mouse–IgG–FAL serum and pNPP. *B,* glycotope characterization of anti-native hemocyanin antibodies. The same protocol performed in *A* was developed with the sera from mice preincubated with a mix of glycans (mannose and galactose, 100 mm) and then with each hemocyanin. *C,* glycotope characterization of anti-*N*-deglycosylated hemocyanin antibodies. The assay was conducted similar to *A* with the corresponding antisera. *D,* effect of *N*-deglycosylation of hemocyanins on their carrier effect. The anti-DNFB titers of C57BL/6 mice immunized with 0.8 mg of native (*N*) or *N*-deglycosylated (*D*) hemocyanins coupled to DNFB were determined by ELISA using BSA-DNFB and revealed with an anti-mouse–IgG–FAL serum and pNPP. *E,* schedule of the antitumor experiment. Five to seven mice per group were primed with 400 μg of native and *N*-deglycosylated hemocyanins or PBS as a vehicle control. Two weeks later, they were challenged with B16F10 cells. In the following 12 days, 6 doses of 100 μg of hemocyanin were administered as immunotherapy. *F,* effect of *N*-deglycosylation of hemocyanins on the tumor growth. Tumor size was measured every 3 days until day 20 previous to the exponential growth of the tumor. Tumor volume was calculated using the ellipsoid formula. *G,* effect of *N*-deglycosylation of hemocyanins on survival of mice. Survival rates were followed up to 45 days. For all *graphs,* the results are representative of two independent experiments. Data are shown as the mean ± S.E. Analysis by *t* test. *, *p* < 0.05; **, *p* < 0.01.

Hemocyanins are widely used molecules as adjuvants and carriers, whose reliable effects have been proved in several models. Therefore, we aimed to investigate the effect of hemocyanin *N-*glycans on some of their biomedical and biotechnological applications. First, we assessed the effect of *N-*deglycosylation on the humoral response induced by CCH, FLH, and KLH. Thus, C57BL/6 mice were immunized with 1 mg of each native and *N-*deglycosylated hemocyanin or with PBS alone as vehicle control, and serum antibodies against each hemocyanin and their respective *N-*deglycosylated form were analyzed by ELISA. We observed that native FLH and KLH showed the highest specific antibody titers (Fig. 6*A*) than their *N-*deglycosylated forms, whereas CCH showed no differences in both conditions. Additionally, we observed that the incubation of each sera with a mix of monosaccharides (mannose and galactose) diminished their binding to native hemocyanins but not to *N-*deglycosylated hemocyanins (Fig. 6, *B* and *C*), suggesting that a significant number of antibodies recognize glycotopes of hemocyanins as described previously for KLH (44). Subsequently, we studied the effect of hemocyanin *N-*deglycosylation on their carrier properties because these proteins are widely used to produce antibodies against peptides, hapten molecules, and tumor-associated antigens (1). For this purpose, we immunized C57BL/6 mice with native or *N-*deglycosylated hemocyanins coupled to the hapten DNFB, and serum antibodies against BSA-DNFB were analyzed (Fig. 6*D*). Results showed that *N-*deglycosylation significantly decreased the carrier properties of FLH, as the anti-DNFB antibody titer was significantly diminished, compared with the native protein. For KLH, we observed a clear decreasing trend, whereas with CCH no significant differences were found.

Finally, the antitumor effects of hemocyanins have been studied in several models, such as superficial bladder cancer, melanoma, and oral cancer (37–39), among others. Interestingly, the B16F10 murine melanoma model described by Arancibia *et al.* (6) shows a significantly attenuated antitumor effect of chemically-deglycosylated FLH, compared with the native protein. Thus, we used the abovementioned melanoma model to assess the effect of *N-*deglycosylation on the antitumor effects of CCH, FLH, and KLH. The schedule of the bioassay is shown in Fig. 6*E*. C57BL/6 mice (seven mice per group) were primed with each native and *N-*deglycosylated hemocyanin or with PBS alone as vehicle control. After 14 days, all mice were subcutaneously challenged with 2 × 10^5^ B16F10 cells. On days 1–12, mice received six doses of native or *N-*deglycosylated hemocyanin as immunotherapy. Using this model, we confirmed the antitumor properties of CCH, FLH, and KLH, which significantly decreased tumor volume and increased survival rates compared with those of the PBS group (Fig. 6, *F* and *G*). *N-*Deglycosylated CCH and FLH showed no significant difference in tumor volume, neither with native proteins nor with the PBS group. However, the *N-*deglycosylated KLH group showed a smaller tumor volume than the PBS group but a significantly higher tumor volume than the native KLH group, indicating a diminished antitumor effect. Otherwise, we observed no significant differences in the survival rates between the native and *N-*deglycosylated groups. However, for FLH and KLH, *N-*deglycosylation tended to decrease survival rates. The median survival of the native and *N-*deglycosylated FLH groups was 27 and 23 days, respectively, whereas the median survival of the PBS group was 20 days. Similarly, the median survival of the native and *N-*deglycosylated KLH groups was 31 and 27 days, respectively. Moreover, the *N-*deglycosylated group reached 100% death, whereas the native KLH group reached only 86% death when the experiment was finished on day 45. Taken together, these results show the major effects of *N-*deglycosylation in the immunomodulatory effects of hemocyanins. The humoral response induced by FLH and KLH as well as the carrier effect of FLH were significantly affected after *N-*glycan removal. However, the antitumor effect of KLH was significantly diminished, even though the treatment with PNGase F did not eliminate *O-*linked oligosaccharides, such as Thomsen-Friedenreich (Fig. 1), which play a role in tumor progression and tumor invasiveness (71).

In conclusion, these data demonstrated that the *N-*glycan content of hemocyanins is, among other structural characteristics, an important factor required for their immunological activity.

## Discussion

Mollusk hemocyanins are widely-distributed glycoproteins. A large and compelling body of evidence has revealed the beneficial immunological effects of hemocyanins in biomedicine; however, the molecular and cellular bases of their immunostimulatory activities remain unclear. Here, we clarified the role of oligosaccharides in the structure and immunogenic potential of CCH, FLH, and KLH. Previous studies have already suggested the participation of glycans in the immune response induced by hemocyanins as chemically-deglycosylated hemocyanins show differences in cytokine secretion and antitumor effects compared with the native proteins (6, 28, 30). Nevertheless, chemical deglycosylation protocols have restrictions, as many glycans are resistant to this treatment. Indeed, up to 80% of mannoses and galactoses from fetuin are resistant to sodium periodate oxidation (45). Additionally, this method leads to the oxidation of amino acid residues forming Schiff bases that promote their cross-linking and stabilize their didecameric structure (6, 34). Thus, the effects of sugar–protein cross-linking and the effects of a simple loss of the glycans on the protein cannot be differentiated. Although the aforementioned studies have helped elucidate the role of hemocyanin glycans, these limitations made it necessary to develop an alternative method to support the results from previous studies.

Asparagine-linked sugar attachment, known as *N-*glycosylation, is one of the most common post-translational modifications in glycoproteins, and enzymatic deglycosylation with PNGase F is one of the most popular methods for this purpose (46). Hemocyanin glycosylation sites are located on the external side of the didecamers and also in oxygen-binding sites or between FUs (16), which are not accessible to the enzyme. Published PNGase F protocols suggest denaturing or reducing proteins to achieve extensive glycan removal in these conditions; however, CCH, FLH, and KLH formed aggregates upon denaturation or reduction. Hence, in this study, we used a modified protocol of enzymatic *N-*deglycosylation by PNGase F treatment. Hemocyanin subunits were gently dissociated to maintain protein solubility despite the significant amount of remnant glycans. Otherwise, we expected a partial deglycosylation, as KLH contains glycans that cannot be removed by PNGaseF, such as fucose(α1–3)-GlcNAc (20, 22, 25).

Our results contributed to characterize hemocyanin monosaccharides, as complex centers of GlcNAc composed of more than 3 units of this moiety, α-mannose and fucose, in complex branches were detected. β-Galactose signals were identified in FLH and KLH, whereas GalNAc was detected in only FLH. Both signals decreased after PNGase F treatment, confirming the presence of galactose-containing *N-*glycans (20). These results obtained by lectin array blotting confirmed the findings of putative glycan branches previously detected by MS (6). Strikingly, the presence of the T antigen was detected in all hemocyanins. Such complex and heterogeneous carbohydrates indicate that hemocyanins could interact with several CLRs as multivalent ligands, producing their extensive effects. Similarly, the results provided insight into the percentage of FLH *N-*glycosylations (∼4.1% w/w), which had not been previously estimated. Thus, FLH has more abundant and heterogeneous glycosylations than CCH and KLH, which is concordant with its higher immunostimulatory and antitumor potential (6, 36).

Furthermore, our results support the structural role of hemocyanin *N-*glycans, as *N-*deglycosylation impaired quaternary structure, consistent with previous studies (16). Indeed, glycosylations reduce chemical and physical protein instabilities as they increase the internal noncovalent forces and the solvent-accessible surface area, among other parameters. The effects of glycosylation on protein stability are highly sensitive to the location of the glycosylation site in the tridimensional structure of the proteins (47–49). In the case of hemocyanins, some *N-*glycans are located at the C-terminal end of FUs, at the interface between subunits (16). Thus, binding between subunits, which is normally reinforced by glycans, would be decreased by *N-*deglycosylation. Similarly, our results showed that *N-*deglycosylation impaired FLH and KLH refolding. As described, oligosaccharides might modulate the local protein structure, as well as its vicinity, contributing to the interactions near their attachment sites (50–53). Therefore, *N-*deglycosylated hemocyanins might form lesser stable intermediate structures than native hemocyanins, slowing the refolding process and refolding to dissimilar structures. Consequently, these conformationally altered species might interact with themselves, possibly leading to additional physical instabilities, such as aggregation, as observed for CCH (52). Nevertheless, we could not determine whether *N-*deglycosylation impaired hemocyanin tertiary structure and whether this effect contributes to the decrease in didecameric structures. In contrast, hemocyanin *N-*deglycosylation had no effect on the secondary structure. It is important to note that the number of glycans attached can apparently impart different stabilization effects on the protein structure. Indeed, a few *N-* and *O-*oligosaccharides, such as remnant glycans, would be sufficient to stabilize the secondary structure of many proteins (51–53). These results were also expected because the noncovalent interactions, such as hydrogen bonds, are able to maintain the stability of the secondary structure (51, 52).

In this work, we demonstrated the binding of CCH, FLH, and KLH to MR, MGL, and TLR4. Our previous studies shown that TLR4 contributes to the hemocyanin immune response; thus, its binding to hemocyanins was used as a control (30). The binding of hemocyanins to the abovementioned receptors might partially explain their immunological effects. Indeed, highly-mannosylated ligands of MR are efficiently presented to T lymphocytes in both MHC-I and MHC-II (54); however, MR lacks a signaling motif and requires assistance from other receptors (55). Remarkably, the binding of hemocyanins to MGL had not been described until the present work. Hence, the binding of FLH and KLH to MGL, consistent with the results of lectin array blotting analyses, might also contribute to their immune responses, favoring their endocytosis and subsequent antigenic presentation (56). Interestingly, chemically-deglycosylated FLH and KLH showed increased binding to MGL, probably because this protocol removes only some external glycans, leaving exposed remnant galactoses as described previously (45). Thus, similar or increased binding of enzymatically or chemically deglycosylated FLH and KLH to MGL might contribute to explain their remnant immunomodulatory effects. Indeed, recent studies using the arthropodan *Litopenaeus vannamei* hemocyanin, a galactose-rich protein with beneficial immunostimulant and antimicrobial effects, have confirmed that its beneficial effects are promoted by its galactose-rich *O-*glycosylations (57). It is important to note that in this study we assessed hemocyanin binding to MR, MGL, and TLR4; however, as these proteins are multiligand molecules they might bind to several other immune receptors, such as the glycan-recognizing DC-SIGN or TLR2. Although these receptors require further investigation, our current preliminary studies suggest their contribution to hemocyanin immunomodulatory effects (28, 30).

Glycan-dependent hemocyanin binding to innate immune receptors has immunological consequences, such as increased production of proinflammatory cytokines (35). The results from J774.2 macrophages showed diminished secretion of TNFα, IL-6, and IL-12p40 induced by *N-*deglycosylated FLH and KLH compared with native hemocyanins. Thus, decreased binding to receptors correlates with a reduced proinflammatory response of APCs. Decreased production of TNFα could diminish systemic immune responses; however, impaired production of IL-6 and IL-12 could lead to a reduced humoral response and/or a diminished Th1 adaptive response (58–60). As demonstrated previously, *N-*deglycosylated hemocyanins have remnant mannoses and fucoses; the latter are also capable of binding to innate immune receptors, inducing alternative Nf-κB activation pathways (60). Therefore, we cannot eliminate the possibility that *N-*deglycosylated hemocyanins would induce alternative proinflammatory pathways, in which this transcription factor is involved.

Additionally, glycan-dependent hemocyanin binding to innate immune receptors promotes their endocytosis in APCs (34). To demonstrate that *N-*glycans contributed to this process, we partially inhibited macropinocytosis in J774.2 macrophages prior to incubation with hemocyanins. In this condition, we observed a partial decrease in the binding and incorporation of *N-*deglycosylated FLH and KLH, although it was not abrogated. This might be considered a significant effect, as DMA produced a marked but partial inhibition of macropinocytosis. Hence, hemocyanins would still be incorporated in a nonspecific manner. Moreover, macropinocytosis is only one of several mechanisms described for antigen incorporation, such as clathrin- or caveolin-dependent pathways, that are not inhibited by DMA treatment and would also contribute to hemocyanin incorporation in a glycan-independent manner (61–64). Thus, considering that there are multiple specific and nonspecific endocytosis pathways in APCs, the differences observed in binding and uptake of FLH and KLH after *N-*deglycosylation suggest that glycans might contribute to some extent to this process.

To analyze the participation of *N-*glycans on the biomedical and biotechnological applications of hemocyanins, we analyzed the effect of *N-*deglycosylation on the humoral response and on the carrier properties of hemocyanins, in a model where hemocyanins were used as carriers to produce antibodies against a hapten (DNFB). We observed that *N-*deglycosylated FLH and KLH showed a reduced humoral response, readout by a lower antibody titer against hemocyanins, compared with that of native proteins. These results suggest that hemocyanin carbohydrates participate in the specific humoral response, which is mediated by B lymphocytes that recognize various antigens, including oligosaccharides (65, 66). Indeed, anti-glycan antibodies have been previously reported; for instance, infections by some glycosylated parasites, such as *Schistosoma mansoni*, *Toxocara canis*, and *Hemonchus contortus*, among others, efficiently induce anti-glycan IgM and IgG (66, 67). Actually, specific serum against CCH, FLH, and KLH shows a decreased reactivity against hemocyanins in the presence of soluble mannose and galactose. Thus, a significant number of antibodies recognize hemocyanin glycotopes. Of note, when serum against *N-*deglycosylated hemocyanins were incubated with the same mix of glycans, their reactivity did not change. As glycoproteins contain many glycotopes, *N-*deglycosylation of hemocyanins may reduce the number of these antigenic determinants, and therefore, the mice immunized with them have decreased specific anti-glycan antibodies in the serum. In addition, a reduction in the glycotopes in *N-*deglycosylated hemocyanins could result in decreased stimulation of innate immune receptors, which would eventually lead to diminished cytokine production, as shown previously, and this change could inhibit the proinflammatory environment, decreasing the hemocyanin presentation to helper T lymphocytes, as hemocyanins are T-dependent antigens (68). In addition, *N-*deglycosylation of FLH impaired its carrier properties using DNFB as a hapten molecule. For KLH, we observed a decreasing trend in the carrier effect after *N-*deglycosylation, whereas CCH did not show significant differences, which is consistent with the previous results. Therefore, the carrier–hapten complex formed by *N-*deglycosylated FLH or KLH and DNFB might be less immunogenic, compared with the native protein, which correlates with the decreased hemocyanin immunostimulant effects after glycan removal.

Additionally, we performed a bioassay using a B16F10 murine melanoma model and analyzed the effect of *N-*deglycosylated hemocyanins on the antitumor response. Hemocyanins have shown antitumor properties by themselves in several *in vitro* and *in vivo* models of cancer. Our results showed partially diminished antitumor effects induced by *N-*deglycosylated FLH and KLH compared with native proteins. These effects could be related to the decreased antibody titer and diminished IL-12 induced by *N-*deglycosylated FLH and KLH, which could lead to diminished IFN-γ levels and impaired cytotoxic responses (69, 70). It is important to consider that treatment with PNGase F does not eliminate all sugars, and as shown in this work, the hemocyanins also exhibit *O-*linked galactose (*O*-Gal) and GalNAc (*O*-GalNAc) glycans which participate in the antitumor effect, such as T antigen. It has been described that tumor cells often up-regulate *O-*Gal and *O-*GalNAc, which could play key roles in tumor invasiveness and cancer progression (71). Indeed, the antitumor effect of hemocyanins is partially due to the cross-reaction of antibodies that recognize common glycotopes between tumor cells and hemocyanins. Therefore, native hemocyanins, which have both types of sugars, have a major antitumor effect. In this context, the minor level of antibodies against *N-*deglycosylated rather than native hemocyanins also is consistent with the results found (Fig. 6*A*). In addition, the role of *O-*glycans, which were not eliminated from hemocyanins, has been described as a dual effect, as they could bind to lectins and stimulate immune responses, but they could also bind to regulatory cells and promote a tolerogenic effect during tumor growth (72–74).

Native and *N-*deglycosylated CCH did not show any differences in our experiments. Previous surface plasmon resonance analyses demonstrated persistent binding between chemically deglycosylated CCH and chimeric DC-SIGN, whereas deglycosylated FLH and KLH binding was abrogated in these conditions (28). Furthermore, compared with FLH or KLH, CCH has exhibited essential differences in some of its immunomodulatory effects. For instance, previous cytokine analyses have shown that the temporality and intensity of cytokine responses induced by CCH are significantly different from FLH and KLH, whose responses were more similar to LPS than CCH. Moreover, quantitative PCR analyses demonstrated that the effect of FLH and KLH on cytokine-coding gene expression is not comparable with the effect of CCH (35). Therefore, even though the three hemocyanins induce a specific Th1 immune response, there are essential differences in their temporality and mechanisms involved. These differences are also observed in their structural features. CCH, in contrast to FLH and KLH, is a highly-stable protein, whose melting point is over 100 °C (15). Moreover, CCH does not require bivalent cations to preserve its heterodidecameric structure, in contrast to KLH and FLH, which form homodidecameric structures dependent on calcium and magnesium (5). Therefore, these essential differences in CCH, compared with FLH and KLH as epitopes, could be essential for the contribution of *N-*glycan to its immune responses.

Taken together, these results help elucidate the relationship between the structure and immunomodulatory mechanisms of hemocyanins. In addition, we confirmed that *N-*glycosylations can lead to enhanced molecular stabilities and therapeutic efficacies of protein pharmaceuticals (18, 75, 76), such as FLH and KLH. Indeed, *N-*glycans contribute to their structural stability, and as shown here, when the oligosaccharides were partially removed, their immunogenicity was reduced. Thus, hemocyanin glycans should be considered in the development of new biomedical and biotechnological applications of these glycoproteins.

## Experimental procedures

### Ethics statement

Experiments were carried out in accordance with the Guidelines for the Care and Use of Laboratory Animals (National Commission for Scientific and Technological Research of Chile (CONICYT)). The CONICYT Committee on Animal Welfare approved all animal protocols used in this study (FONDECYT 1151337). Animals were monitored daily, and end points to reduce pain or distress were used during the survival study based on the following criteria: physical appearance, behavior, movement, hydration, stool consistency, and tumor volume. Animals were euthanized by cervical dislocation. C57BL/6 mice were obtained from the Universidad de Chile (Santiago, Chile) and housed at 22–24 °C with a light/dark cycle of 12/12 h. Sterile water and food were available *ad libitum*.

### Chemicals

All chemicals were of analytical-grade, and the solutions were prepared using water for human irrigation (Baxter Healthcare, Charlotte, NC, or Fresenius Kabi, Australia) and filtered through a 0.2-mm membrane filter (Millipore, Billerica, MA).

### Hemocyanin sources

Hemocyanins from CCH and FLH were provided by Biosonda Corp. (Santiago, Chile). Hemocyanins were obtained under sterile and pyrogen-free conditions and suspended in phosphate-buffered saline buffer (PBS: 0.1 m sodium phosphate, 0.15 m NaCl, pH 7.2) or Tris buffer (50 mm Tris, pH 7.4, 5 mm CaCl_2_, 5 mm MgCl_2_, and 0.15 mm NaCl). Hemocyanin from *M. crenulata* (KLH) suspended in PBS was purchased from Calbiochem (Merck, Darmstadt, Germany). OVA was purchased from InvivoGen. Dissociated hemocyanins were produced by dialysis against dissociation buffer (130 mm glycine containing 2.5 mm EDTA, pH 9.6) (5).

For *N-*deglycosylation of hemocyanins and OVA, different protocols using PNGase F (Asparia Glycomics, San Sebastian, Spain) were assessed according to manufacturer's instructions and as described by Freeze and Kranz (46). First, hemocyanins were heated at 100 °C for 5 min in denaturing buffer (0.5 m sodium bicarbonate buffer, 0.25% SDS, and 50 mm β-mercaptoethanol, pH 7.5). Then, 0.25% Triton® X-100 and 2 μl of PNGase F in deionized water were added and incubated at 37 °C for 24 h. Alternatively, 1 mg of each hemocyanin was dialyzed against dissociation buffer (130 mm glycine containing 2.5 mm EDTA, pH 8.6), and then heated at 60 or 100 °C for 15 min. Hemocyanins were cooled to room temperature, and 2 μl of PNGase F in deionized water were added and incubated for 24 h at 37 °C. As the first protocol removed most *N-*glycans but decreased protein solubility, the second protocol was used for the subsequent experiments.

Chemically deglycosylated hemocyanins and OVA were produced by treatment with sodium periodate using the protocol described by Arancibia *et al.* (6). After both deglycosylation methods, proteins were dialyzed against PBS or Tris buffer containing 5 mm MgCl_2_ and 5 mm CaCl_2_, pH 7.2, in Amicon Ultra-15 10K MWCO tubes (Millipore). The final protein concentration was determined by the Pierce® 660-nm colorimetric method (Thermo Fisher Scientific) according to the manufacturer's instructions.

### Glycan analyses

Sugar moieties in native and *N-*deglycosylated hemocyanins, as well as in OVA, were analyzed by lectin array blotting from Asparia Glycomics (77). Briefly, hemocyanins (0.5 mg/ml) were labeled with Alexa Fluor-555–NHS dye and subsequently diluted in PBS with 0.5% Tween 20, 0.4 mg/ml BSA, and 0.3 mm CaCl_2_. Labeled proteins were incubated for 1 h at room temperature in glass sheets previously prepared with 48 different lectins. Finally, the glass sheets were washed with PBS and scanned with a G265BA Microarray Scanner (Agilent Technologies). The data obtained were analyzed with Proscan Array Express software (PerkinElmer Life Sciences).

In addition, hemocyanin glycans were analyzed by dot blot using Pierce® glycoprotein staining kit (PAS staining, Thermo Fisher Scientific) according to the manufacturer's instructions. Additional analyses were performed using ConA staining (Thermo Fisher Scientific). For the dot blots, 30 μg of protein were incubated with ConA (2 μg/ml) on a nitrocellulose membrane and then with avidin coupled to alkaline phosphatase (avidin–AP) (1:1,000) and were revealed using the colorimetric reagent NBT-BCIP (Thermo Fisher Scientific). Membranes were scanned and analyzed with the ImageJ software. For ELISA analyses, proteins (10 μg/ml) were incubated on 96-well polystyrene plates with ConA (2 μg/ml) and then with avidin–AP (1:1.000) and revealed using the colorimetric reagent *p-*nitrophenyl phosphate (pNPP) (Thermo Fisher Scientific) (1 mg/ml) in the FAL buffer (100 mm Tris-HCl, 100 mm NaCl, 1 mm MgCl_2_, pH 9.5) at 37 °C. Optical density was measured at 405 nm on a Synergy HTX ELISA plate reader (Biotek).

### SDS-PAGE

Acrylamide gels (5–15%) were prepared according to the Laemmli method (78). Briefly, proteins were heated at 100 °C for 5 min in the presence of SDS and β-mercaptoethanol. Electrophoresis was performed in running buffer (0.1 m Tris, 0.1 m HEPES, 3 mm SDS, pH 8.0) at 60 V for 1 h and at 100 V for 3 h. Gels were stained with Coomassie Blue. The molecular weight standard Kaleidoscope^TM^ Precision Plus Protein (Bio-Rad) was used in all experiments.

### CD

CD analyses were performed as described by Kelly *et al.* (79). Briefly, the spectra of each hemocyanin were measured in the far-UV region (200–260 nm) in a model J-1500 JASCO spectropolarimeter (JASCO, Japan) of the Facultad de Ciencias Químicas y Farmacéuticas from the Universidad de Chile (Santiago, Chile). For all experiments, hemocyanin samples were analyzed at a concentration of 0.25 mg/ml in 0.1-cm trays with an optical passage at 25 °C. For the analysis of reduced hemocyanins, proteins were incubated with 2% β-mercaptoethanol. To generate unfolding curves, hemocyanins were incubated with increasing concentrations of GdmHCl (0–6.7 m) (Sigma) for 24 h. For the refolding curves, hemocyanins were incubated for 24 h with 6.7 m GdmHCl and then diluted in PBS to obtain different concentrations of GdmHCl (0.5–6.7 m). For all samples, spectra were taken between 200 and 260 nm, and similarly, the dichroism signal was measured at 222 nm for 1 min.

### TEM

Hemocyanin samples (0.6 mg/ml) in parlodion-coated copper grids were stained with 1–2% uranyl acetate for 30 s. The grids were dried at room temperature and analyzed using a Phillips Tecnai 12 electron microscope at the Microscopy Service of Pontificia Universidad Católica de Chile (Santiago, Chile). Approximately 20 representative images of different samples and different fields were taken at 80 kV with increases of ×60,000 and ×87,000, and didecamers, decamers, and dissociated structures were counted.

### SEC

Chromatography was performed on a TSKgel® G5000PWXL column (10 μm, 7.8 mm inner diameter × 30 cm) (TOSOH, Japan) at room temperature (25 °C) according to the manufacturer's instructions. The column was pre-equilibrated in PBS, pH 7.2, and then native, dissociated, and *N-*deglycosylated hemocyanins (1 mg/ml) were loaded in PBS, pH 7.2, at 0.6 ml/min. Chromatography was monitored at 280 nm. The molar extinction coefficients used for estimating the amount of protein were 1.4 for CCH and 2.02 for FLH and KLH according to the manufacturer's instructions. Each fraction was collected and concentrated using Amicon Ultra-15 10K MWCO tubes (Millipore). Further analyses were performed by SDS-PAGE and by ConA staining as described previously.

### Hemocyanin binding assay to chimeric receptors

Binding assays were performed by ELISA according to the general procedure described by Royer *et al.* (80). Briefly, polystyrene plates were activated with native, dissociated, or deglycosylated hemocyanins (10 μg/ml) overnight at 4 °C. Furfurman (10 μg/ml) (cell wall extract from *Malassezia furfur*) (Invivogen), lipopolysaccharide (LPS; 1 μg/ml) from *Escherichia coli* (Enzo Life Sciences, New York), and galactose (100 mm) (Alfa Aesar) were used as positive controls. Gelatin (Merck) was used as a negative control. Hemocyanins were incubated with the chimeric receptors MR-Fc (Luisa Martínez-Pomares Laboratory, University of Nottingham, UK), MGL-Fc (Abcam, Cambridge, UK), and TLR4-Fc (R&D Systems, Minneapolis, MN) (1 μg/ml) and diluted in TBS buffer (10 mm Tris-HCl, 10 mm CaCl_2_, 154 mm NaCl, and 0.05% (v/v) Tween 20, pH 7.5) for 2 h at room temperature. Next, plates were incubated with goat anti-human IgG serum (Sigma) coupled to FAL (1:1.000) in TBS for 1 h at room temperature. Between each step, the plates were washed three times with TBS. Binding was revealed by adding pNPP (Thermo Fisher Scientific) (1 mg/ml) to the FAL buffer at 37 °C. Optical density was measured at 405 nm on a Synergy HTX ELISA plate reader (Biotek).

For competitive ELISAs, analyses were performed as described above, but each receptor was incubated with d-(+)-mannose (Sigma) or d-(+)-galactose (Alfa Aesar) (0–100 mm) for 30 min, prior to their binding to hemocyanins.

### Fluorescent-labeled hemocyanins

Hemocyanins were labeled with DyLight–NHS-488 reagent (Thermo Fisher Scientific) according to the manufacturer's instructions. Briefly, 1 m bicarbonate was added to the hemocyanins until a pH of 8.5 was reached. The hemocyanins and staining reagent were mixed at a 1:7 ratio. The mixture was incubated at room temperature for 1 h, and the reaction was stopped with the addition of hydroxylamine, pH 6.5. The hemocyanins were dialyzed against PBS, pH 7.2, and stored in the dark at 4 °C until use.

### J774.2 macrophage cell line culture

Mouse macrophages (J7742 cell line) were cultured at 37 °C in a 5% CO_2_ atmosphere. RPMI 1640 culture medium was supplemented with 10% fetal bovine serum (FBS), 2 mm l-glutamine, 1 mm pyruvate sodium, 1 mm nonessential amino acid solution minimum Eagle's medium, and 1 mm penicillin and streptomycin (all from HyClone). For the experiments, cells were harvested by incubation with 0.25% trypsin and 0.1% EDTA (HyClone) for 10 min. The cell count was performed using a Neubauer camera.

### Analyses of CLR expression in J774.2 macrophages

J774.2 cells were incubated with primary antibodies in PBS with 2% FBS, including anti-MGL, anti-TLR4 (Abcam), and anti-MR (Biolegend), in addition to the rat isotype IgG2a control (Abcam) (1:100), at 4 °C for 1 h. Then the cells were incubated with secondary antibody (goat anti-rat IgG-FITC serum, Thermo Fisher Scientific) (1:1,000) or with conjugated anti-F4/80–Alexa Fluor-647 (Biolegend) antibody for 30 min in the dark. The cells were washed with 2% PBS/FBS between each step. Finally, the cells were fixed with PBS/paraformaldehyde (PBS/PFA, 2%), and the tubes were incubated at 4 °C in the dark. Data were acquired in a BD FACSort cytometer from the Facultad de Ciencias, Universidad de Chile, and subsequently processed using FlowJo 6.0 software.

### Hemocyanin binding/incorporation by J774.2 cells

J774.2 cells (2,5–5 × 10^5^), seeded in 24-well plates, were incubated with DMA (Sigma) (200 μg/ml) in serum-free culture medium for 30 min at 37 °C. Next, the cells were incubated with native or *N-*deglycosylated hemocyanins conjugated to Alexa Fluor-488 (50 μg/ml) for 1 h at 37 °C. For inhibition assays, cells were previously incubated with d-(+)-mannose (Sigma) or d-(+)-galactose (Alfa Aesar) (100 mm) and then incubated with hemocyanins as described above. Subsequently, the cells were incubated with an eFluor 780 viability probe (Thermo Fisher Scientific) (1:1,000) for 30 min at 4 °C. Cells were fixed with 2% PBS/PFA, and data were acquired in a BD FACSort cytometer from the Facultad de Ciencias, Universidad de Chile and subsequently processed using FlowJo 6.0 software.

### Cytokine quantification

J774.2 cells (5 × 10^5^) were incubated with native, dissociated, and *N-*deglycosylated hemocyanins (500 μg/ml) in serum-free culture medium for 24 h at 37 °C as described by Jiménez *et al.* (30). As a positive control, cells were incubated with LPS from *E. coli* (Enzo Life Sciences) (100 ng/ml). As a negative control, cells were incubated with culture medium without hemocyanin. Culture supernatants were collected and quantified by ELISA using BD OptEIA^TM^ kits (BD Biosciences) to detect IL-6, IL-12p40, and TNFα, according to the manufacturer's instructions.

### B16F10 melanoma cell line culture

The murine melanoma cell line B16F10 was cultured at 37 °C in a 5% CO_2_ atmosphere in RPMI 1640 medium containing 10% FBS, 2 mm l-glutamine, 1 mm sodium pyruvate, 1 mm solution of nonessential amino acids minimal essential medium, and 1 mm penicillin and streptomycin (all from HyClone). For the experiments, cells were harvested with 0.25% trypsin and 0.1% EDTA (HyClone) for 5 min. Cells were counted using a Neubauer camera.

### Antitumor assay

Mouse melanoma assays were performed according to the protocol described by Arancibia *et al.* (6) with modifications. Briefly, C57BL/6 mice (five or seven animals per group for the first or second bioassay, respectively) were primed intraperitoneally with 400 μg of native or *N-*deglycosylated hemocyanin. A control group was primed only with PBS, which was the hemocyanin vehicle. Two weeks later, on day 0 of the experiment, all groups were challenged with 1.5 × 10^5^ B16F10 cells for the first bioassay and with 2 × 10^5^ B16F10 cells for the second bioassay, in RPMI 1640 medium with 2% FBS by subcutaneous injection. Therapeutic doses of 100 μg of native or *N-*deglycosylated hemocyanin were administered subcutaneously on days 1, 3, 5, 7, 9, and 12 of the experiment. The occurrence of tumors and their dimensions, as well as the health status of the experimental animals, were monitored visually and by palpation every 3 days. The tumor volume was estimated by measuring the length and width of the tumor and subsequently using the ellipsoid formula ((length × width × width)/2). On day 25 of the first bioassay, the animals were bled to obtain the immune sera. These samples were analyzed to quantify the antibody titer, as described below.

### Anti-hemocyanin humoral immune response assessment by ELISA

The specific antibody titer was determined as described by Arancibia *et al.* (6). Briefly, ELISA plates were activated with each hemocyanin (10 μg/ml), blocked with PBS with 1% casein (Sigma) for 1 h at room temperature, and incubated for 90 min at 37 °C with 50 μl of the corresponding serum. Then, plates were incubated with goat anti-murine IgG antibody (Thermo Fisher Scientific) (1:2,500) for 30 min at 37 °C and revealed using 1 mg/ml pNPP in FAL buffer. Between each stage, the plates were washed with 1% casein PBS. Optical density was measured at 405 nm in the Synergy HTX ELISA plate reader (BioTek). The antibody titer was defined operationally as the dilution of the serum at which half the maximum absorbance was obtained.

### Hemocyanin coupling to DNFB

5 mg of each native or *N-*deglycosylated hemocyanin was diluted in 0.5 m NaHCO_3_ to a final volume of 1 ml. Next, 25 μl of DNFB was added, and the mixes were incubated at 37 °C for 45 min. Finally, each sample was dialyzed against PBS buffer, and the recovered proteins were quantified by Bradford 660 colorimetric assay.

### Carrier effect of hemocyanins

C57BL/6 mice (three animals per group) were immunized intraperitoneally with 400 μg of native or *N-*deglycosylated hemocyanins, alone or coupled to DNFB, on days 0 and 10. A control group was primed only with PBS, which was the hemocyanin vehicle. Two weeks later, the animals were bled to obtain the immune sera. To analyze the anti-DNFB antibody titer, ELISAs were performed using the same previously described protocol for estimating anti-hemocyanin antibodies, but each 96-well polystyrene plate was activated with BSA-DNFB (10 μg/ml).

### Statistical analyses

Most of the experiments were performed at least twice independently, in duplicate or triplicate when possible. The results were plotted using GraphPad Prism 6 and analyzed by Student's *t* test or by one- or two-way ANOVA, as indicated in each case.

## Author contributions

M. L. S., J. M. J., J. V., M. B., A. M., and M. I. B. conceptualization; M. L. S., J. M. J., J. V., M. R., M. B., A. M., and M. I. B. formal analysis; M. L. S. and M. I. B. validation; M. L. S., M. R., and M. I. B. investigation; M. L. S. and M. I. B. visualization; M. L. S., M. R., M. B., and M. I. B. methodology; M. L. S. and M. I. B. writing-original draft; M. L. S., J. M. J., J. V., M. R., M. B., A. M., and M. I. B. writing-review and editing; J. M. J., M. R., M. B., A. M., and M. I. B. supervision; M. I. B. resources; M. I. B. data curation; M. I. B. funding acquisition; M. I. B. project administration.

## Supplementary Material

Supporting Information

## Footnotes

3 The abbreviations used are:

KLH: *M. crenulata* hemocyanin
PNGase F: peptide:*N*-glycosidase F
CCH: *C. concholepas* hemocyanin
FLH: *F. latimarginata* hemocyanin
FU: functional unit
MR: mannose receptor
TLR4: Toll-like receptor 4
MGL: macrophage galactose lectin
APC: antigen-presenting cell
GdmHCl: guanidinium hydrochloride
NBT-BCIP: nitro blue tetrazolium–5-bromo-4-chloro-3-indolyl phosphate
PAS: periodic acid-Schiff
OVA: ovalbumin
ANOVA: analysis of variance
TEM: transmission EM
pNPP: *p-*nitrophenyl phosphate
DNFB: 1-fluoro-2,4-dinitrobenzene
FBS: fetal bovine serum
SEC: size-exclusion chromatography
TLR: Toll-like receptor
CLR: c-type lectin receptor
DC-SIGN: dendritic cell-specific intercellular adhesion molecule-3–grabbing nonintegrin
ConA: concanavalin A
DMA: *N,N*-dimethyl amiloride
AP: alkaline phosphatase
PFA: paraformaldehyde
MFI: mean fluorescence intensity
LEL: lycopersicon esculentum lectin
RCA: ricinus communis agglutinin
STL: solanum tuberosum lectin
PSA: pisum sativum agglutinin
ACA: amarantus caudatus lectin.
