# Supplementary material for: N-Glycosylation of mollusk hemocyanins contributes to their structural stability and immunomodulatory properties in mammals
Source: J Biol Chem. 2019 Nov 12;294(51):19546–64. doi: 10.1074/jbc.RA119.009525 (PMC6926458; doi:10.1074/jbc.RA119.009525)
Supplement: Supporting Information [file supp_294_51_19546__index.html]

N-glycosylation of mollusk hemocyanins contributes to their structural stability and immunomodulatory properties in mammals — Structural and immunogenic hemocyanin N-glycosylations role — N-Glycosylation of mollusk hemocyanins contributes to their structural stability and immunomodulatory properties in mammals — Structural and immunogenic hemocyanin N-glycosylation role — Supporting Information 

# *N*-Glycosylation of mollusk hemocyanins contributes to their structural stability and immunomodulatory properties in mammals

## Supporting Information

- Supplementary figures and tables - Table S1. Estimated percentage of N-glycans removed from CCH, FLH and KLH. Table S2. Lectins used for lectin array blotting assays. Figure S1. Quaternary didecameric structure of native, chemically deglycosylated and dissociated hemocyanins. Figure S2. Dissociation did not affect hemocyanin binding to chimeric innate immune receptors and cytokine secretion in J774.2 macrophages. Figure S3. Kinetic of cytokine secretion of J774.2 macrophages induced by native or N-deglycosylated hemocyanins.
