# Supplementary material for: N-Glycosylation of mollusk hemocyanins contributes to their structural stability and immunomodulatory properties in mammals
Source: J Biol Chem. 2019 Nov 12;294(51):19546–64. doi: 10.1074/jbc.RA119.009525 (PMC6926458; doi:10.1074/jbc.RA119.009525)
Supplement: Supporting Information [file supp_RA119.009525_153106_2_supp_426756_q0t3x3.pdf]

N-glycosylations contribute to the structural stability and immunomodulatory properties of mollusk hemocyanins in mammals

Michelle L. Salazar[1], José M. Jiménez[1], Javiera Villar[1], Maira Rivera[2], Mauricio Báez[2], Augusto Manubens[3], María Inés Becker[1,3*]

[1]Fundación Ciencia y Tecnología para el Desarrollo (FUCITED). Santiago, Chile.
[2]Departamento de Bioquímica y Biología Molecular, Facultad de Ciencias Químicas y Farmacéuticas, Universidad de Chile, Santiago, Chile.
[3]Departamento de Investigación y Desarrollo, Biosonda Corp. Santiago, Chile

Running title: *Structural and immunogenic hemocyanin N-glycosylations role*

Supplementary material:

Table S1. Estimated percentage of N-glycans removed from CCH, FLH and KLH.

Table S2. Lectins used for lectin array blotting assays.

Figure S1. Quaternary didecameric structure of native, chemically deglycosylated and dissociated hemocyanins.

Figure S2. Dissociation did not affect hemocyanin binding to chimeric innate immune receptors and cytokine secretion in J774.2 macrophages.

Figure S3. Kinetic of cytokine secretion of J774.2 macrophages induced by native or N-deglycosylated hemocyanins.

Table S1. Estimated percentage of N-glycans removed from CCH, FLH and KLH

Hemocyanin	Reported glycan percentage (w/w)	Estimated N-glycan percentage (w/w)
CCH	3.1	2.5 ± 0.42
FLH	ND	4.10 ± 1.12
KLH	3.4	2.3 ± 0.42

The electrophoretic migration of the native and N-deglycosylated hemocyanins was estimated, and differences in molecular weight were quantified from at least 3 different gels. From this difference, the percentage (w/w) of removed N-glycans was estimated. Percentages are shown as mean±SD. ND: not determined.

Table S2. Lectins used for lectin array blotting assays

Monosaccharide	Lectin	Specificity
Mannose (Man)	Concanavalin A (ConA)	α-Man, α-Glc 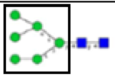
	Narcissus pseudonarcissus lectin (NPL)	Internal and terminal Man 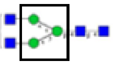
	Hippeastrum hybrid agglutinin (HHA)	α-1,3Man, α-1,6Man 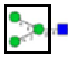
N-Acetilglucosamine (GlcNAc)		(GlcNAc)-Sial 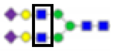
	Triticum vulgaris agglutinin (WGA)	
	Lycopersicon esculentum lectin (LEL)	(GlcNAc)3 - GlcNAc 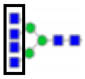
	Solanum tuberosum lectin (STL)	(GlcNAc)3, LacNAc-GlcNac 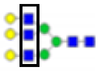
Galactose (Gal) and N-Acetilactosamine (LacNAc)	*Ricinus communis* agglutinin (RCA)	β-Gal, Lac, LacNAc 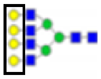
	Phaseolus Vulgaris agglutining (PHA)	LacNAc oligosaccharides 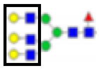
	Cicer arietinum lectin (CAL)	Lactose 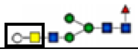
Fucose (Fuc)	*Pisum sativum* agglutining (PSA)	Fuc α-1,6 -GlcNAc and α-Man-Fuc 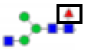
	Lens culinaris agglutining (LCA)	-Man/GlcNAc(α-1,6)-Fuc 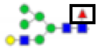
T antigen	*Vicia villosa* B4 lectin (VVL)	GalNAc-GalNac 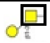
	Agaricus bisporus lectin (ABL)	α-Gal(1-3)GalNAc
	Amarantus caudatus agglutining (ACA)	T antigen 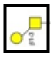
	Maclura pomifera agglutining (MPA)	T antigen
N-Acetilgalactosamine (GalNAc)	*Soybean agglutinin* (SBAmax)	α-Gal-GalNAc 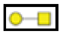
	Arachis hypogaea agglutinin (AHA)	Gal-β-1,3 - GalNAc
	Wisteria floribunda lectin (WFL)	GalNAc 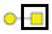

Specificity of each lectin used for recognizing the following monosaccharides: mannose (green circles), N-acetylglucosamine (blue squares), galactose (yellow circles), fucose (red triangles) and N-acetylgalactosamine (yellow squares). The monosaccharides recognized in glycosylation branches are shown in black squares in order to highlight lectin specificity.

Figure S1

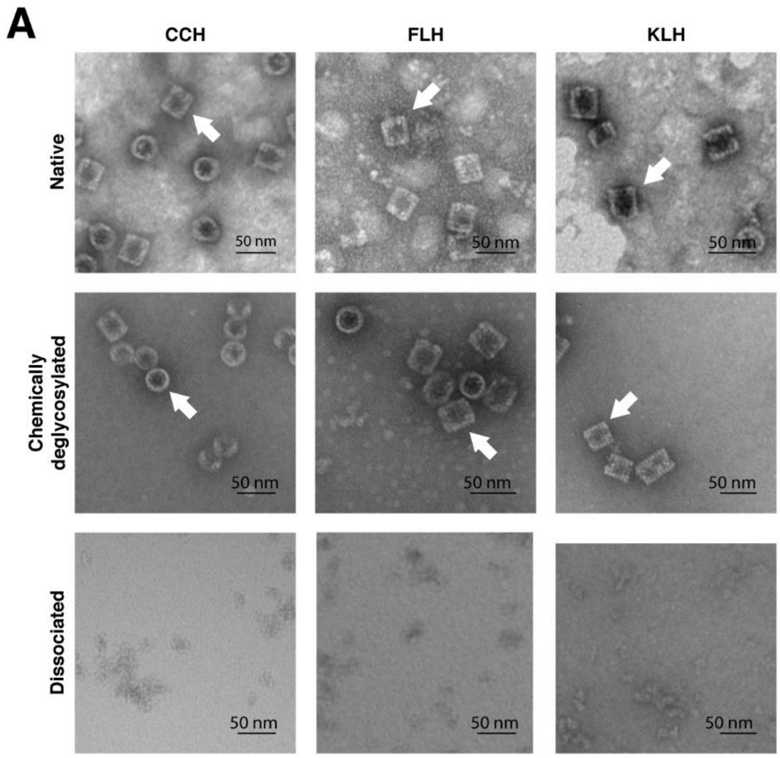

Figure S1. Quaternary didecameric structure of native, chemically deglycosylated and dissociated hemocyanins. Analysis by transmission electron microscopy with negative staining. Representative images of each hemocyanin at 60,000x, in which didecamers (white arrows) are observed. Native hemocyanin photographs are already used in Figure 2A. Scale bar: 50 nm.

Figure S2

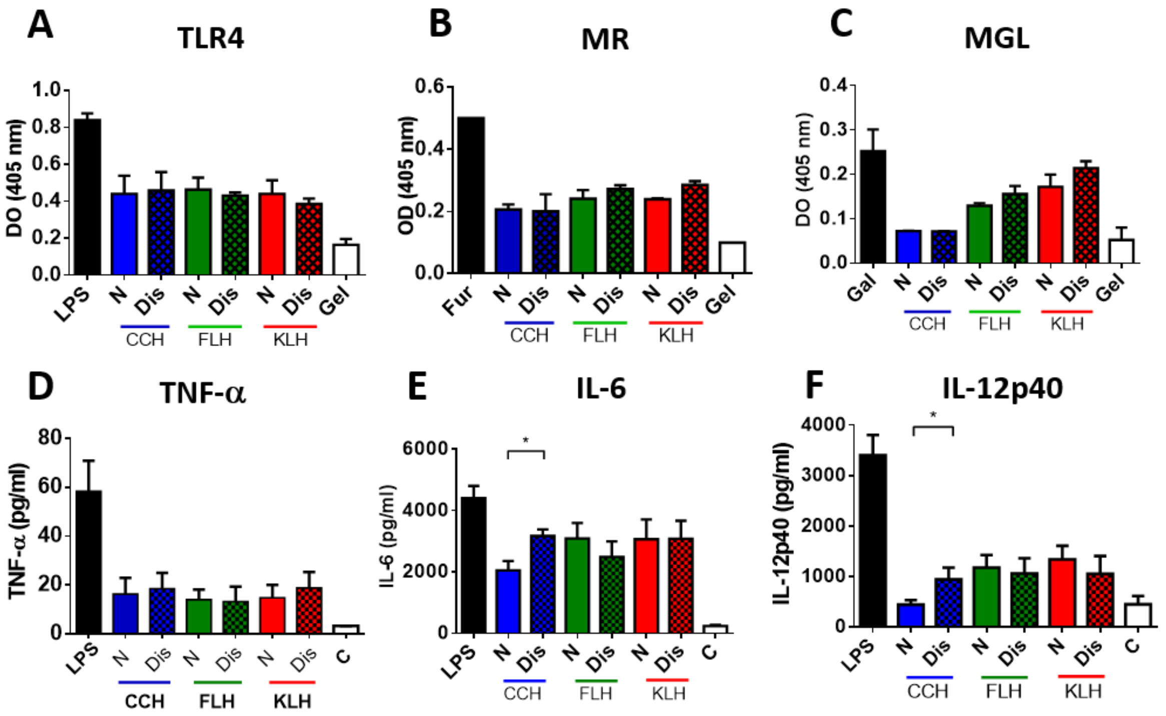

Figure S2. Dissociation did not affect hemocyanin binding to chimeric innate immune receptors and cytokine secretion in J774.2 macrophages. Native (N) and dissociated (Dis) hemocyanins (2.5 µg/ml) were incubated with the chimeric receptors MR **(A),** MGL **(B)** and TLR4 **(C)** (1 µg/ml) and with anti-Fc antibodies. Furfurman (Fur), lipopolysaccharide from *E. coli* (LPS) and D-(+)-galactose (Gal) were used as positive controls, whereas gelatin (Gel) was the negative control. Additionally, TNF-α **(D)**, IL-6 **(E)** and IL-12p40 **(F)** from J774.2 culture supernatants were quantified by ELISA after 24 hours of incubation with native (N) and Dissociated (Dis) hemocyanins. LPS from *E. coli* and the culture medium without hemocyanin (C) were used as positive and negative controls, respectively. For all experiments, the data are shown as the mean±SEM of three independent experiments. Analyses by T test. *p<0.5. **p<0.01. ***p<0.001. ****p<0.0001.

Figure S3

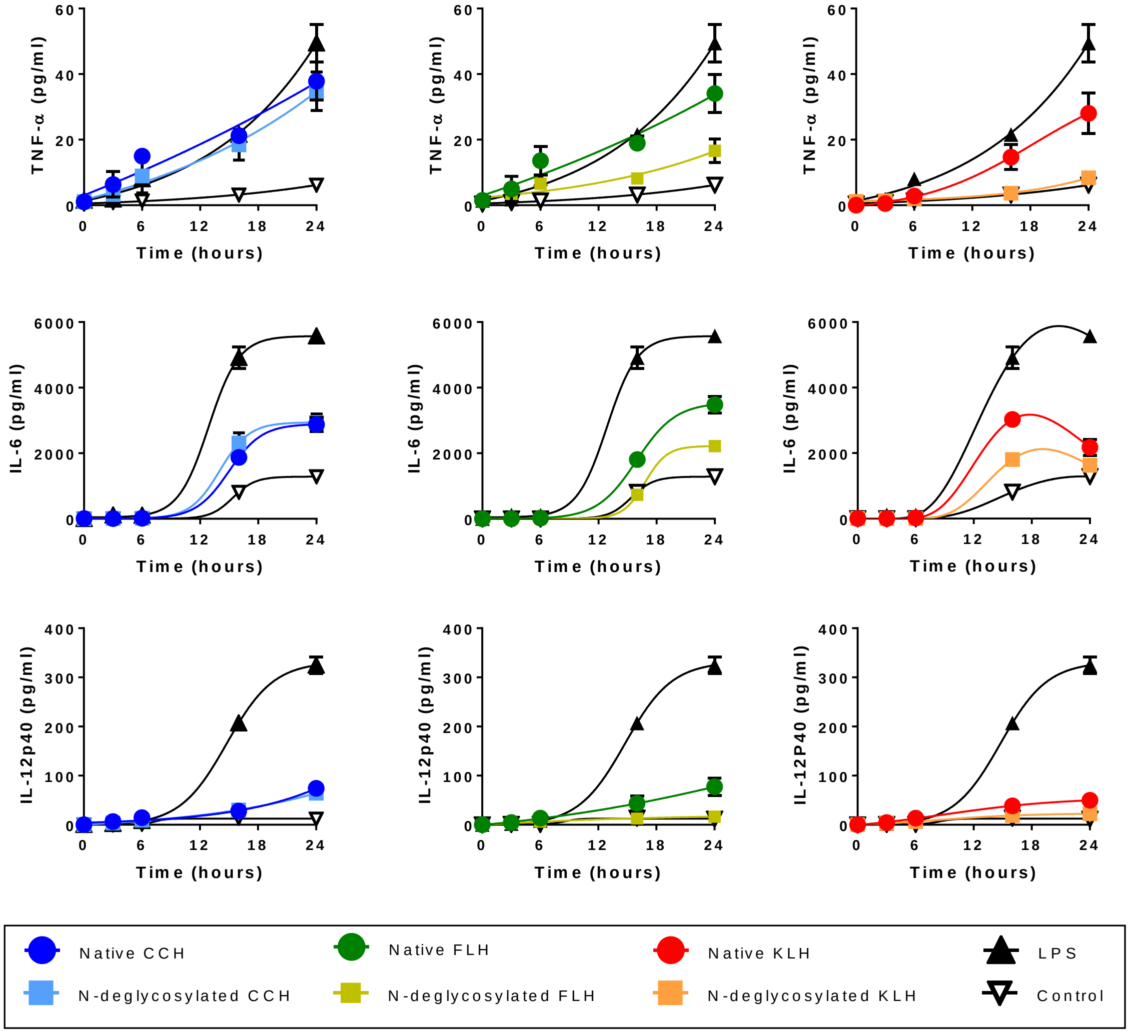

Figure S3. Kinetic of cytokine secretion of J774.2 macrophages induced by native or N-deglycosylated hemocyanins. TNF-α, IL-6 and IL-12p40 from J774.2 culture supernatants were quantified by ELISA at 0, 6, 18 and 24 hours of incubation with the native (N) or N-deglycosylated (D) hemocyanins. LPS from *E. coli* and the culture medium without hemocyanin (C) were used as positive and negative controls, respectively. For all experiments, the data are shown as the mean±SEM of three independent experiments. Analyses by T test. *$p<0.5$. **$p<0.01$. ***$p<0.001$. ****$p<0.0001$.
